# Fear avoidance and catastrophizing are associated with both knee awareness and quality of life in knee osteoarthritis patients: a secondary report of a cross-sectional study

**DOI:** 10.2340/17453674.2025.45070

**Published:** 2025-12-18

**Authors:** Nina Jullum KISE, Siri ELIASSEN, Ove FURNES, Caryl GAY, Stig HEIR, Anners LERDAL, Maren Falch LINDBERG, Turid ROGNSVÅG, Arild AAMODT, Tor Kjetil NERHUS

**Affiliations:** 1 Department of Orthopedic Surgery, Martina Hansens Hospital, Baerum, Norway; 2 Department of Physiotherapy, Martina Hansens Hospital, Baerum, Norway; 3 The Norwegian Arthroplasty Register, Department of Orthopedic Surgery, Haukeland University Hospital, Bergen, Norway; 4 Department of Clinical Medicine, University of Bergen, Norway; 5 Department of Family Health Care Nursing, University of California, San Francisco, CA, USA; 6 Research Department, Lovisenberg Diaconal Hospital, Oslo, Norway; 7 Department of Public Health Science and Interdisciplinary Health Sciences, Institute of Health and Society, Faculty of Medicine, University of Oslo, Oslo, Norway; 8 Department of Orthopedic Surgery, Lovisenberg Diaconal Hospital, Oslo, Norway; 9 Coastal Hospital in Hagevik, Department of Orthopedic Surgery, Haukeland University Hospital, Bergen, Norway; 10 Department of Clinical Medicine, University of Bergen, Bergen, Norway

## Abstract

**Background and purpose:**

In patients with knee osteoarthritis (OA), psychological factors (anxiety, depression, and pain-related catastrophizing) are associated with more pain and worse physical function. Low knee awareness and high knee-related quality of life (QoL) are key indicators of a well-functioning knee. The objective of our study was to evaluate associations between psychological factors and knee awareness and knee-related QoL in patients with knee OA.

**Methods:**

In this Norwegian cross-sectional study of 653 patients with knee OA, 4 psychological factors were assessed: anxiety, depression, pain-related catastrophizing, and fear avoidance of physical activity. Associations between these factors and knee awareness and knee-related QoL were examined in unadjusted and adjusted regression models, controlling for age, sex, BMI, pain, and whether patients accepted or declined inclusion in a randomized controlled trial (ClinicalTrials.gov: NCT03771430). Regression coefficients with values below zero indicate negative associations between the independent and dependent factors and values above zero indicate positive associations.

**Results:**

Worse scores on all 4 psychological measures were associated with higher knee awareness and poorer knee-related QoL in unadjusted analyses. Standardized estimates (βs) ranged from –0.38 (95% confidence intervals [CI] –0.45 to –0.31) to –0.16 (CI –0.23 to –0.08). In adjusted analyses, pain catastrophizing (β –0.07, CI –0.14 to –0.01) and fear-avoidance (β –0.11, CI –0.18 to –0.05) remained associated with higher knee awareness, whereas poorer knee-related QoL remained associated with more anxiety (β –0.10, CI –0.16 to –0.03) and depression (β –0.14, CI –0.20 to –0.08), as well as more pain catastrophizing (β –0.19, CI –0.26 to –0.12) and fear-avoidance (β –0.19, CI –0.25 to –0.13).

**Conclusion:**

Higher fear avoidance of physical activity and more pain catastrophizing had the strongest associations with higher knee awareness and poorer knee-related QoL.

Pain is among the most frequently reported symptoms in patients with osteoarthritis (OA) [1].

Psychological factors like anxiety, depression [2], and pain-related catastrophizing have been associated with more pain and lower physical function [3]. Poor outcomes in OA patients could partially be explained by similar processes to those observed in a subset of patients with acute low back pain who develop chronic pain due to pain-related fear of movement [4].

Pain reduction is an important goal in the treatment of OA, but 2 other key indicators of a well-functioning knee with OA are low knee awareness, where patients can almost forget about their arthritic joint in everyday life [5], and high knee-related quality of life (QoL) [6]. Knee awareness in OA is a relatively new concept and is not only considered a measure of presurgical knee function, but low knee awareness has also become the ultimate goal of achieving a natural-feeling knee following total knee arthroplasty (TKA) [7].

Research on relevant psychological factors and their associations with knee awareness and knee-related QoL in OA patients is scarce, despite prior evidence of these factors’ importance [3].

Such knowledge is critically needed for individually tailored treatment interventions, where the focus must be on treating the patients holistically, taking into account mental and social factors rather than only the physical symptoms of the disease. For example, it is possible that some knee OA patients may benefit from additional patient education or psychological treatment to mitigate psychological factors, such as fear avoidance and pain catastrophizing. If these factors are associated with knee awareness and knee-related QoL, psychological treatment may also contribute to better results after surgery.

To address these gaps in knowledge, this cross-sectional study’s objective was to evaluate the concurrent relationships between psychological factors (pain-related catastrophizing, fear of movement, anxiety and depression) and both knee awareness and knee-related QoL in patients with knee OA. The hypotheses were that a heavier burden of these psychological factors was associated with both more knee awareness and poorer knee-related QoL.

## Methods

This study is a sub-study of the MultiKnee trial (i.e., “Multidisciplinary Intervention in Total Knee Arthroplasty trial”), a 3-armed multi-center randomized controlled trial (RCT) [8]. Included knee OA patients were assigned to the MultiKnee program (OA education, exercise therapy, and cognitive behavioral therapy) either with or without supplementary TKA, or to the control group, who received the usual TKA treatment without the Multiknee program. Patients assigned to the MultiKnee program without supplementary TKA were asked to postpone the surgery for at least 1 year following treatment [8]. This cross-sectional study is reported according to the STROBE guidelines for observational studies [9].

This study includes preoperative data for 653 patients with knee OA eligible for TKA. The total sample included both patients who participated in the RCT (n = 280) and patients who declined the RCT but agreed to participate in an observational study with similar measures (n = 373); the study the patients participated in is referred to as their “inclusion status.”

Patients were recruited from the 3 largest knee centers in Norway: Lovisenberg Diaconal Hospital (LDS), Coastal Hospital in Hagevik (CiH), and Martina Hansen’s Hospital (MHH). Recruitment started in September 2020 and finished in October 2023.

### Patient selection

All patients in this study were considered by an orthopedic surgeon to have an indication for TKA, and all were evaluated and approved for surgery. The inclusion criteria were age 18–79 years, a body mass index (BMI) less than 40, a Kellgren–Lawrence (KL) OA radiologic grade [10] of 3 or 4, American Society of Anesthesiologists Physical Status classification (ASA) grade of 1–3, and the ability to read and communicate in Norwegian. Exclusion criteria were previous unicompartmental or patellofemoral arthroplasty in the index knee, large axis deviations requiring use of a hinged prosthesis, diagnosis of dementia, or diagnosis of a chronic inflammatory joint disease (e.g., rheumatoid arthritis and Bechterew’s disease).

### Data collection

Patient-reported data were collected and stored electronically using the Service for Sensitive Data at University of Oslo [11]. The questionnaires included demographic and clinical characteristics (e.g., age, sex, and BMI) and reliable and validated patient-reported outcome measures (PROMs).

### PROMs: KOOS and FJS

Knee-related pain and QoL were assessed using the Pain and QoL subscales of the Knee Injury and Osteoarthritis Outcome Score (KOOS) [12]. KOOS is a 42-item survey to assess people’s perceived difficulties with activity due to problems with their knees during the past week [12]. Each of the 42 items carries equal weighting (0–4), with higher scores indicating worse outcomes. KOOS has 5 subscales: pain, other symptoms, activities of daily living (ADL), function in sport and recreation, and knee-related QoL. For each subscale, the scores are reversed and transformed to a 0–100 scale, with 0 indicating extreme knee problems and 100 indicating no problems [12]. The Pain subscale has 9 items assessing pain intensity in various daily activities, and the QoL subscale has 4 items assessing the frequency of knee problems, whether patients can rely on their knee, whether they have changed their lifestyle, and how much trouble they have with their knee [12]. The QoL subscale has been shown to perform particularly well in capturing aggregate knee-specific outcomes in knee OA patients [13].

Knee awareness was assessed with the Forgotten Joint Score (FJS) [5]. The FJS measures patients’ knee awareness, or the ability to forget about a joint in one’s everyday life [5]. Patients rate their agreement with 12 statements on a scale that ranges from 0 (never) to 4 (mostly). The raw score is transformed to a 0–100 score and then reversed to obtain the final score. Higher scores are better, as they indicate less knee awareness. FJS was originally designed as a tool for measuring postoperative outcomes following TKA [5]. It is shown to have good construct validity and test–retest reliability, low ceiling and floor effects, and is a valuable tool in discriminating between patients with good outcomes vs excellent outcomes [14].

### PROMS: Psychological factors

4 psychological factors were assessed: pain-related catastrophizing with the Pain Catastrophizing Scale (PCS) [15], fear avoidance of physical activity with the Fear-Avoidance Belief Questionnaire (FABQ) physical activity subscale [16], and symptoms of anxiety and depression with the 2 subscales of the Hospital Anxiety and Depression Scale (HADS) [17]. The PCS consists of 13 items that assess 3 dimensions of catastrophizing (i.e., rumination, magnification, helplessness) and can also be summed to a total score ranging from 0–52, with higher scores indicating more catastrophizing [15]. The FABQ consists of 2 subscales, fear-avoidance beliefs for work and physical activity [16], but only 1, the physical activity subscale, consisting of 4 items, was used in this study. Scores range from 0–24, and higher scores indicate more avoidance. The HADS consists of 14 items, 7 on the anxiety subscale and 7 on the depression subscale. Subscale scores range from 0–21, with higher scores indicating more anxiety or depression. Scores < 8 suggest no anxiety or depression, and scores > 8 suggest mild to severe anxiety or depression [17]. The Norwegian version has excellent psychometric properties [18].

### Statistics

The statistical methods have been prepared in line with the recommendations of Acta Orthopaedica [19]. The data was analyzed using IBM SPSS statistical software version 29.0 (IBM Corp, Armonk, NY, USA). Descriptive data is presented as means and standard deviations (SD) for continuous variables or as counts and percentages for categorical variables. Simple and multiple linear regression models were used to assess both unadjusted and adjusted associations between the 4 psychological factors (including the 3 subdimensions of the PCS) (independent variables) and both the knee awareness and knee-related QoL measures (dependent variables). The multiple regression models adjusted for the following possible confounders: age, sex, BMI, knee pain (measured with the KOOS Pain subscale), and RCT inclusion status (whether the patients were included in the MultiKnee trial or had declined). The 3 demographic factors and knee pain were included due to their presumed impact on the outcome measures. RCT inclusion status was included because we assumed that there could be a difference in psychological factors between patients who take the chance of being included in a study with a 1 in 3 risk of having to wait a year for a planned TKA, and a 2 in 3 risk of being assigned to cognitive behavioral therapy.

The regression coefficients were presented as unstandardized (B) and standardized (β) estimates, both with 95% confidence intervals (CI), and the significance level was set to P = 0.05. Coefficients with values below zero indicate negative associations between the independent and dependent factors, values of zero indicates no associations, and values above zero indicate positive associations. The unstandardized estimates (B) indicate how much the dependent variable changes when the independent variable changes by 1 point. The standardized estimates (β) indicate the relative strength of the associations between the different independent variables and the dependent variables and can therefore be considered effect sizes. A total of 14 unadjusted and 14 adjusted regression models were evaluated, but due to the exploratory nature of this study no adjustments for multiple testing were performed [20].

### Ethics, data sharing plan, funding, use of AI, and disclosures

All patients provided their electronic consent for inclusion. The MultiKnee trial (including both the RCT and the observational study) was performed according to the Declaration of Helsinki, approved by the Ethics Committee of South-Eastern Norway Regional Health Authority (2017/968), and registered in ClinicalTrials.gov (NCT03771430). AI tools were not used.

TG had a PhD grant from the Western Norway Regional Health Authority (#912219). AL and AA are funded by the Research Council of Norway (#287816). MFL is funded by the South-Eastern Norway Regional Health Authority (#2022007). The MultiKnee trial is supported by the Research Council of Norway (#287816).

Each author certifies that he or she has no commercial associations (e.g., consultancies, stock ownership, equity interest, patent/licensing arrangements, etc.) that might pose a conflict of interest in connection with the submitted article. Complete disclosure of interest forms according to ICMJE are available on the article page, doi: 10.2340/17453674.2025.45070

## Results

**Figure 1:**
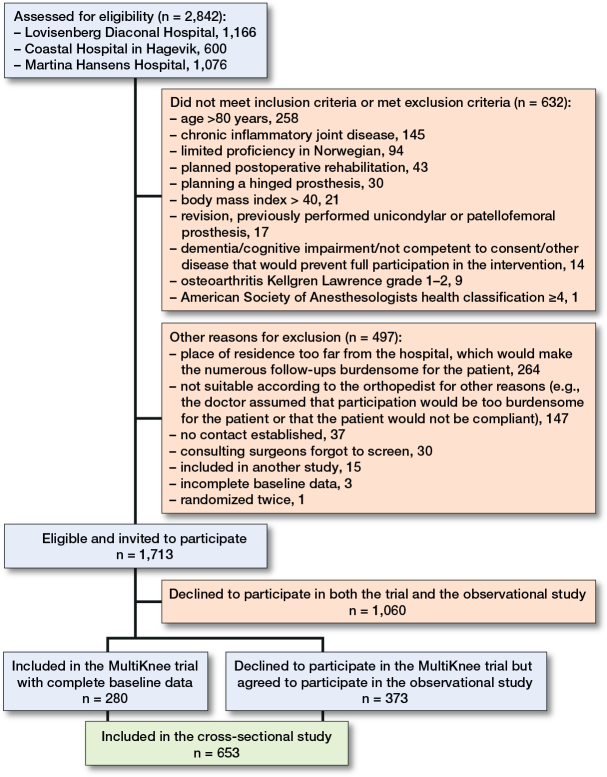
Flowchart of included participants with knee osteoarthritis evaluated to have indication for knee replacement.

Of 2,842 patients assessed for eligibility, 1,713 were invited to participate in the MultiKnee trial. 280 accepted to participate and 373 declined but accepted to participate in the observational study (Figure 1). Hence, 653 patients were included in this study. The patients did not differ by hospital on age, sex, or BMI. The demographic characteristics and scores on measures of knee-related pain, psychological factors, knee-awareness, and knee-related QoL for the total sample showed that 40% of the patients were men, mean age was 67.1 years (SD 7.7), and mean BMI was 28.6 (SD 4.4). Mean HADS Anxiety and Depression were 3.6 (SD 3.2) and 3.2 (SD 2.7) points, respectively, mean PCS Total score was 14.3 points (SD 10.6), and mean FABQ Physical activity subscale score was 12.6 points (SD 6.3). Mean KOOS QoL score was 28.1 points (SD 13.8) and mean FJS was 16.2 points (SD 13.6) (Table 1).

**Table 1:** Demographic characteristics, psychological factors, knee-related QoL, and knee-awareness scores in osteoarthritis patients with indication for knee replacement (N = 653). Values are mean (standard deviation) unless otherwise specified

Variables	
Hospital, n (%)	
Lovisenberg Diaconal Hospital	242 (37)
Coastal Hospital in Hagevik	197 (30)
Martina Hansens Hospital	214 (33)
Age	67.1 (7.7)
Male sex, n (%)	260 (40)
Body mass index	28.6 (4.4)
HADS subscale scores	
Anxiety	3.6 (3.2)
Depression	3.2 (2.7)
HADS subscales, dichotomized, n (%)	
Anxiety	
< 8 (no anxiety)	580 (88)
≥ 8 (mild to severe anxiety)	77 (12)
Depression	
< 8 (no depression)	599 (91)
≥ 8 (mild to severe depression)	58 (8.8)
PCS total and subscale scores	
Total	14.3 (10.6)
Rumination	4.8 (3.8)
Magnification	2.4 (2.3)
Helplessness	7.1 (5.3)
FABQ physical activity subscale	12.6 (6.3)
KOOS subscale scores	
Symptoms	48.8 (18.0)
Pain	45.2 (16.7)
Activities of daily living	55.5 (18.3)
Sport & recreation	16.4 (15.4)
Quality of life	28.1 (13.8)
Forgotten Joint Score	16.2 (13.6)

HADS: Hospital Anxiety and Depression Scale (range 0–21, higher = more anxiety/depression), PCS: Pain Catastrophizing Scale (total: range 0–52, higher score = more catastrophizing; subscale ranges: rumination: 0–16, magnification: 0–12. helplessness: 0–24), FABQ: Fear-Avoidance Belief Questionnaire (range 0–24, higher = more avoidance), KOOS: Knee injury and Osteoarthritis Outcome Score (range 0–100, higher = better), Forgotten Joint Score (range 0–100, higher = better).

In unadjusted analyses, worse scores for each of the 4 psychological factors (anxiety, depression, pain-related catastrophizing, and fear avoidance of physical activity) were associated with higher knee awareness and poorer knee-related QoL. Standardized estimates (βs) ranged from –0.38 (CI –0.45 to –0.31) to –0.16 (CI –0.23 to –0.08) (Table 2).

**Table 2:** Unadjusted and adjusted regression analyses of psychological variables’ associations with FJS and KOOS-QoL

Independent variables	Unadjusted (simple) linear regression models		Adjusted (multiple) linear regression models	
	B (CI)	ß (CI)	B (CI)	ß (CI)
**Models with FJS as dependent variable**				
HADS Anxiety	–0.68 (–1.01 to –0.35)	–0.16 (–0.23 to –0.08)	–0.25 (–0.52 to 0.01)	–0.06 (–0.12 to –0.00)
HADS Depression	–0.79 (–1.17 to –0.41)	–0.16 (–0.23 to –0.09)	–0.27 (–0.58 to 0.03)	–0.06 (–0.11 to 0.00)
FABQ Physical Activity Scale	–0.47 (–0.63 to –0.31)	–0.22 (–0.30 to –0.13)	–0.23 (–0.37 to –0.10)	–0.11 (–0.18 to –0.05)
PCS Total score	–0.36 (–0.45 to –0.26)	–0.28 (–0.35 to –0.20)	–0.09 (–0.18 to –0.01)	–0.07 (–0.14 to –0.01)
PCS Rumination subscore	–0.92 (–1.19 to –0.70)	–0.26 (–0.34 to –0.19)	–0.22 (–0.44 to 0.01)	–0.06 (–0.12 to 0.00)
PCS Magnification subscore	–1.00 (–1.45 to –0.55)	–0.17 (–0.23 to –0.10)	–0.17 (–0.54 to 0.21)	–0.03 (–0.09 to 0.03)
PCS Helplessness subscore	–0.75 (–0.94 to –0.56)	–0.30 (–0.36 to –0.23)	–0.22 (–0.06 to –0.39)	–0.09 (–0.15 to –0.02)
**Models with KOOS–QoL as dependent variable**				
HADS Anxiety	–0.85 (–1.18 to –0.51)	–0.19 (–0.27 to –0.11)	–0.42 (–0.70 to –0.15)	–0.10 (–0.16 to –0.03)
HADS Depression	–1.23 (–1.60 to –0.85)	–0.24 (–0.31 to –0.17)	–0.73 (–1.04 to –0.42)	–0.14 (–0.20 to –0.08)
FABQ Physical Activity Scale	–0.65 (–0.81 to –0.49)	–0.30 (–0.37 to –0.22)	–0.42 (–0.56 to –0.28)	–0.19 (–0.25 to –0.13)
PCS Total score	–0.48 (–0.58 to –0.39)	–0.37 (–0.44 to –0.30)	–0.25 (–0.33 to –0.16)	–0.19 (–0.26 to –0.12)
PCS Rumination subscore	–1.19 (–1.45 to –0.92)	–0.33 (–0.40 to –0.26)	–0.54 (–0.77 to –0.31)	–0.15 (–0.23 to –0.08)
PCS Magnification subscore	–1.60 (–2.05 to –1.16)	–0.27 (–0.33 to –0.20)	–0.84 (–1.22 to –0.46)	–0.14 (–0.20 to –0.08)
PCS Helplessness subscore	–1.00 (–1.18 to –0.82)	–0.38 (–0.45 to –0.31)	–0.52 (–0.70 to –0.35)	–0.20 (–0.28 to –0.13)

All multiple linear regression models adjusted for age, sex, BMI, KOOS–Pain, and inclusion status (whether the patient was included in the MultiKnee trial or declined and participated in the observational study instead).

Abbreviations: See Table 1 and CI: 95% confidence interval.

**Figure 2:**
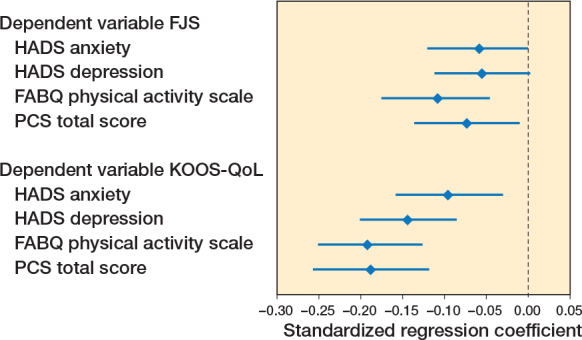
Forest plots of the standardized regression coefficients (diamonds) with 95% confidence intervals (whiskers). Values below zero indicates that higher levels of the dependent variables are associated with inferior levels of the dependent variables. Abbreviations: FJS: Forgotten Joint Score, KOOS-QoL: Quality of Life subscore of the Knee Injury and Osteoarthritis Outcome Score, HADS: Hospital Anxiety and Depression Scale, FABQ: Fear-Avoidance Belief Questionnaire, PCS: Pain Catastrophizing Scale.

When controlling for age, sex, BMI, knee pain, and RCT inclusion status in the adjusted analyses, only more pain catastrophizing (β –0.07, CI –0.14 to –0.01) and more fear-avoidance (β –0.11, CI –0.18 to –0.05) remained associated with more knee awareness (see Table 2). In contrast, more anxiety (β –0.10, CI –0.16 to –0.03) and more depression (β –0.14, CI –0.20 to –0.08), as well as more pain catastrophizing (β –0.19, CI –0.26 to –0.12) and more fear-avoidance (β –0.19, CI –0.25 to –0.13), remained associated with poorer knee-related QoL in the adjusted analyses (Table 2, Figure 2).

In unadjusted analyses of the PCS subscales, more rumination, magnification, and helplessness were associated with more knee awareness and worse knee-related QoL, ranging from β –0.17 (CI –0.23 to –0.10) to β –0.38 (CI –0.45 to –0.31). In the adjusted analyses controlling for age, sex, BMI, knee pain, and inclusion status, statistically significant associations remained between PCS helplessness and knee awareness (β –0.09, CI –0.15 to –0.02), and between all 3 PCS subscales and knee–related QoL ranging from β –0.14 (CI –0.20 to –0.08) to β –0.20 (CI –0.28 to –0.13) (see Table 2).

## Discussion

The objective of our study was to evaluate associations between psychological factors and knee awareness and knee-related QoL in patients with knee OA. Our study is the first to show that psychological factors, including higher levels of anxiety, depression, pain catastrophizing, and fear-avoidance are associated with worse knee-related QoL in patients with knee OA eligible for knee replacement. Additionally, pain catastrophizing and fear-avoidance are associated with more knee awareness. These associations were evident even when controlling for age, sex, BMI, KOOS-Pain, and inclusion status. We showed a higher fear avoidance of physical activity, and more pain catastrophizing had the strongest associations with both higher knee awareness and poorer knee-related QoL. Although the estimates are small-to-medium overall, all of them have the same direction: worse scores for all psychological factors (fear avoidance, pain catastrophizing, anxiety and depression) are associated with greater knee awareness and poorer quality of life, showing a consistency across findings.

More pain-related fear-avoidance had the strongest association with both higher knee awareness and poorer knee-related QoL in adjusted analyses. The fear-avoidance model of pain [21] implies that catastrophic thoughts and beliefs about pain lead to avoidance behavior—a protective response to prevent further injury. These activity adaptations might be appropriate to prevent further tissue damage after acute injuries, but, in chronic conditions such as OA, avoidance behaviors driven by maladaptive pain-related beliefs can become counter-productive, potentially resulting in increased pain and knee awareness and reduced knee-related QoL. Patients might enter a downward cycle of increased pain, increased fear of movement, decreased physical activity, and long-term disability [22]. Previous studies have identified pain-related catastrophizing as a prognostic factor for chronic pain and poorer functioning following TKA [23-25]. The present study, however, is to our knowledge the first to identify an association between fear-avoidance and knee awareness in OA patients eligible for TKA.

This study also reveals associations between more pain catastrophizing and both more knee awareness and poorer knee-related QoL in OA patients. Further, we found associations between worse anxiety and depression, and poorer knee-related QoL. In a study of patients undergoing TKA or total hip arthroplasties, patients with higher levels of anxiety, depression, or pain catastrophizing had worse preoperative pain [2]. Another study concluded that depression and pain catastrophizing have negative long-term influences on the process of pain development and pain endpoints following surgery and other interventions in patients with OA and other rheumatic diseases [26]. In a systematic review and meta-analysis, Olsen et al. concluded that more pain and more pain catastrophizing preoperatively were correlated with more pain 1 year after TKA [3, 25].

However, in our study of preoperative data, the associations between the psychological factors and the knee awareness and knee-related QoL remained significant in multivariable analyses, controlling for pain levels. This result indicates that the associations between worse psychological factors and poorer knee awareness and QoL are not dependent on pain level alone, but rather are independent risk factors for worse outcomes.

### Strengths

This cross-sectional study of patients with knee OA has a large sample size, and due to the electronic data collection system there is no missing data. The generic PROMs used in this study are reliable and validated, and the disease-specific PROMs used are reliable and validated for patients with knee OA.

The multicenter design with 3 hospitals across 2 health regions in Norway is a major strength that increased the representativeness of the sample.

### Limitations

As only 38% (653 out of 1,713) of those eligible were included, we cannot rule out the possibility of selection bias. However, compared with the Norwegian Arthroplasty registry [27], patients in our study were slightly younger (mean age 67.1 vs 68.8 years) but had a similar sex distribution (60.2% women vs 60.5% men) and BMI (28.6 vs 29.1). These small differences might be explained by the inclusion criteria for this study (age under 80 years, BMI less than 40, OA grade of 3 or 4, and ASA grade 1–3). Further, 12% of the participants had HADS scores indicating mild to severe anxiety. A recent study indicated that 1 in 6 patients experiences meaningful anxiety before undergoing TKA [28]. This might indicate that patients in this study do not differ significantly from other patient populations when it comes to demographic and psychological factors.

In the final regression analyses, we controlled for age, sex, BMI, and KOOS-Pain. The rationale behind this conservative approach was to account for the potentially confounding impacts of sex, age, BMI, and degree of pain on both knee awareness and quality of life. However, for pain catastrophizing, pain could be considered either a confounder—if pain is a driver of both pain catastrophizing and knee outcomes—or a mediator—if pain catastrophizing leads to increased pain, which in turn leads to increased knee awareness.

Moreover, this study sample was composed of patients who had consented to participate in an RCT (n = 280) and patients who declined to participate in the RCT but agreed to participate in an observational study instead (n = 373). We therefore controlled for inclusion status to account for the possibility that choosing or declining to participate in the RCT might be associated with knee awareness or knee-related QoL. By controlling for inclusion status, we also accounted for the likely differences in psychological and pain factors between patients who take the 1 in 3 risk of having to wait a year for a planned TKA and the 1 in 3 risk of being assigned to cognitive behavioral therapy.

According to the usual definitions of effect sizes (small effect size: 0.10–0.29, medium effect size: 0.30–0.49, large effect size: > 0.50), 4 of the psychological factors in the unadjusted analyses with KOOS-QoL as the dependent variable were in the moderate range: FABQ (0.30), PCS total (0.37), PCS rumination (0.33), and PCS helplessness (0.38). All other factors had small effect sizes. In the adjusted analyses of knee-related QoL, all factors remained statistically significant but had small effect sizes after controlling for important variables such as pain and BMI, which are well known to have large impacts on quality of life. Although the adjusted effect sizes were attenuated, possibly due to our conservative approach of controlling for both pain and inclusion status, these findings indicate that the relationships between these psychological factors and knee-related QoL are robust and stable.

The findings were less pronounced in the models with FJS as the dependent variable, where in the unadjusted models only 1 psychological factor (PCS helplessness) had a moderate effect size (0.30) and all other effect sizes were small (ranging from 0.16 to 0.28). In the adjusted models controlling for other factors, only 3 of the psychological variables remained significant, and their effect sizes were very small to small (range 0.07 to 0.11). A possible reason for smaller effect sizes in the models with FJS as the dependent variable is that the FJS was originally designed as a postoperative measuring tool, and in this group of patients with knee OA who had not yet undergone TKA there might be a considerable floor effect. However, the FJS was previously used in a study of patients with posttraumatic knee OA not yet treated surgically, and it demonstrated good psychometric properties and a significant correlation with radiological assessments of OA severity [29].

A final study limitation is that although the FJS is a validated measure in patients with OA, the Norwegian version used in this study has not yet been validated. Despite increasing use of the Norwegian FJS in clinical practice, it is possible that it does not have the same psychometric properties as validated versions of the FJS.

### Conclusions

Our study of patients with knee OA eligible for TKA showed that worse levels of selected psychological factors had associations with both higher knee awareness and poorer knee-related QoL. Higher fear avoidance of physical activity and more pain catastrophizing had the strongest associations with knee awareness and QoL.

*In perspective*, experienced clinicians often believe that they can intuitively sense that an individual patient is likely to have an inferior outcome, even if the specific reason cannot be identified. It is not unlikely that some of what the observant clinician senses is related to the patient’s psychological characteristics. In spite of this, in our experience, psychological factors are not routinely considered in orthopedic clinical practice. Cognitive therapy might be an alternative or complementary intervention, as shown in a recent systematic review and meta-analysis [30]. Further studies are needed to explore causality, possible interventions, and cutoffs for and timing of intervention implementation.

## Notes

NJK, AL, MFL, TKN, AA, and SH conceived and designed the study. NJK, TKN, AA, SH, SE, and TR collected the data. NJK and CLG performed the statistical analyses. NJK drafted the manuscript. All authors had access to all data, participated in analysis and interpretation of the data, revision of the manuscript, and gave final approval of the version to be published.

The authors would like to thank the patients in this study for their participation. Further, they acknowledge Lovisenberg Diaconal Hospital, the Coastal Hospital in Hagevik, and Martina Hansens Hospital for accessibility to the outpatient and physiotherapy clinics.

This paper is a product stemming from the Norwegian research project “The MultiKnee trial”. Dr Maren Falch Lindberg is the Principal Investigator (PI) and Drs Anners Lerdal and Arild Aamodt are Co-PIs. Ingvild Buset Bergvad, Alexander Eikrem-Lüthi, and Turid Rognsvåg are PhD students supervised by the seniors Drs Anners Lerdal, Jon Magnussen, Maren Falch Lindberg, Mona Badawy, Ove Furnes, and Søren T. Skou. The other members of the MultiKnee research team are Drs Caryl L. Gay, Stig Heir, Inger Holm, Nina Jullum Kise, Tor Kjetil Nerhus, Milada C. Småstuen, and Jan Stubberud. Katrine Rutledal, Deputy Director for Lovisenberg User Board, provided user participation.

Handling co-editors: Li Felländer-Tsai and Robin Christensen

*Acta* thanks Siri Heijbel and Lina Holm Ingelsrud for help with peer review of this manuscript.
